# Comparative Metabolomics Analysis of *Gastrodia elata* Blume Different Growth Stages: Insights into Chemical Composition and Bioactivities

**DOI:** 10.3390/metabo16040223

**Published:** 2026-03-30

**Authors:** Guoqiang Zhou, Jiayi An, Yi Li, Zunhan Zhang, Yaru Chang, Guanxiu Xiao

**Affiliations:** 1 College of Biological Sciences and Technology, Yili Normal University, Yining 835000, China; 231002098@ylnu.edu.cn (G.Z.);; 2 College of Life Sciences, Henan Normal University, Xinxiang 453007, China

**Keywords:** *Gastrodia elata* Blume, growth stages, metabolomics, bioactive compounds, antioxidant, enzyme inhibition

## Abstract

Background: *Gastrodia elata* Blume (*GE*) is a valuable traditional Chinese medicine with a wide range of clinical applications, yet the relationship between its developmental stages, phytochemical profiles, and functional properties remains poorly characterized. Methods: In this study, an integrated approach combining chemical assays and UHPLC–Orbitrap–MS/MS-based untargeted metabolomics was employed to characterize three growth stages: Mima (MT, seed tubers), Baima (BT, immature tubers), and Jianma (JT, mature tubers). Results: Multivariate statistical analyses demonstrated clear stage-dependent discrimination in metabolic profiles. A total of 31 differential metabolites were identified, including parishin derivatives, phenolics, amino acids, and organic acids. Specifically, Parishin E, Parishin G, total phenolics, and total flavonoids predominated in the early stage (MT), whereas gastrodin and Parishin C progressively accumulated and peaked in the mature stage (JT). Bioactivity assays revealed that *GE* extracts exhibited significant antioxidant and hypoglycemic (α-glucosidase and α-amylase inhibitory) effects, which followed an initial decrease followed by a subsequent recovery during development. Correlation analysis further established a strong relationship between phenolic-associated metabolites (particularly Parishin E/G/H) and the observed bioactivities. Conclusions: In summary, these findings elucidate the metabolic dynamics of *GE* across developmental stages and provide a scientific basis for optimizing harvest timing and raw-material grading to enhance the functional properties of *GE*-derived products.

## 1. Introduction

*Gastrodia elata* Blume (*GE*), traditionally recognized as a prestigious medicinal and edible plant, has been utilized extensively for the management of convulsions, epilepsy, diabetes mellitus, and paralysis [1,2,3]. Recognizing its long-standing use as a traditional food and medicinal resource, *GE* was officially included in the Chinese government’s “Medicine and Food Homology” catalogue in 2019, highlighting its dual nutritional and therapeutic value [4]. Previous phytochemical investigations have revealed that *GE* contains a variety of bioactive constituents, primarily including phenolic compounds (e.g., gastrodin and p-hydroxybenzyl alcohol), organic acids, and amino acid metabolites [5,6]. These metabolites are considered key contributors to the functional properties of *GE* and may vary during plant development, making it important to investigate their dynamic changes across different growth stages.

The pharmacological efficacy of *GE* is fundamentally dictated by its specialized chemical composition [7]. Accumulating evidence suggests that nutritional and phytochemical profiles undergo significant dynamic shifts during the developmental maturation of various plant tissues [8]. For example, phenolic compounds in olive fruits undergo substantial depletion during ripening, while immature navel oranges possess higher flavonoid content and superior antioxidant properties compared to their mature counterparts [9,10]. Such findings underscore that bioactive metabolite accumulation is a dynamically regulated process governed by specific ontogenetic phases [11]. However, despite the criticality of harvest timing, *GE* research has focused almost exclusively on the characterization of fully mature tubers, largely neglecting the stage-dependent metabolic fluctuations across its developmental continuum. Therefore, elucidating the metabolic signatures and associated bioactivity shifts throughout the maturation of *GE* is essential for its comprehensive valorization and industrial advancement.

Untargeted metabolomics based on high-resolution mass spectrometry has become an important approach for comprehensive characterization of plant metabolic profiles [12]. Among the available analytical platforms, UHPLC–Orbitrap–MS/MS offers significant advantages, including high mass accuracy, high resolution, and strong sensitivity for detecting structurally diverse metabolites [13]. These features make it particularly suitable for the global profiling of secondary metabolites in medicinal plants [14]. In recent years, Orbitrap-based untargeted metabolomics has been widely applied to investigate metabolic variations associated with plant developmental stages, environmental factors, and processing conditions in traditional Chinese medicinal materials [15,16]. Such approaches enable the simultaneous detection of a broad spectrum of metabolites, including phenolic compounds, amino acids, organic acids, and glycosides, thereby providing valuable insights into metabolic dynamics and the biochemical mechanisms underlying the formation of bioactive constituents [17].

To bridge this gap, this study provides a comprehensive metabolomics investigation of *GE* tubers at three critical growth stages: small tubers/Mima (seed tubers, MT), Baima (immature tubers, BT), and Jianma (mature flowering tubers, JT). By employing an untargeted metabolomics approach, we aimed to characterize the significant differences in chemical profiles and identify key differential metabolites driving this developmental continuum. Additionally, the major active components were quantified and correlated with their respective bioactivities (antioxidant capacities and hypoglycemic activities). These findings offer new insights into the metabolic evolution of G. elata during maturation and provide a scientific basis for quality standardization and functional food development.

## 2. Materials and Methods

### 2.1. Materials and Reagents

LC–MS-grade acetonitrile, methanol, and formic acid were obtained from Sigma–Aldrich (St. Louis, MO, USA). Ultrapure water was produced using a Milli-Q purification system (Millipore, Bedford, MA, USA). Reference standards of gastrodin, parishin E, parishin G, parishin C, rutin, and gallic acid were purchased from Chem Faces (Wuhan, China). ABTS•^+^ and DPPH• radical scavenging assay kits were supplied by Jiancheng Bioengineering Institute (Nanjing, China). p-Nitrophenyl-α-D-glucopyranoside (pNPG), α-glucosidase, and α-amylase were acquired from Yuanye Biotechnology (Shanghai, China). All other reagents were of analytical grade or the highest commercially available purity.

### 2.2. Sample Collection and Sample Preparation

*GE* tubers were harvested from a standardized cultivation base in Yiliang County, Zhaotong City, Yunnan Province, China. Representative samples were collected at three distinct developmental stages: Mima (seed tubers, MT), Baima (immature tubers, BT), and Jianma (mature tubers, JT). To minimize individual biological variation, three independent biological replicates were prepared for each stage, with each replicate consisting of tubers pooled from multiple individual plants. Upon harvest, fresh tubers were immediately flash-frozen in liquid nitrogen, transported to the laboratory, and subsequently lyophilized. The dried materials were pulverized using a laboratory mill and passed through a 60-mesh sieve to ensure sample homogeneity. The resulting fine powder was stored at −80 °C until further analysis.

Sample extraction was performed following a previously established protocol with minor modifications [18]. Briefly, 100 mg of homogenized *GE* powder was mixed with 3.0 mL of 70% aqueous methanol (*v*/*v*). For quantification and data normalization, 4-chloro-DL-phenylalanine (30 μg/mL) was employed as the internal standard (IS). The mixture was subjected to ultrasound-assisted extraction in an ice-water bath for 10 min, followed by centrifugation at 12,000 rpm for 10 min at 4 °C. This extraction process was repeated two times for each sample. The supernatants from the three extractions were combined and brought to a final volume of 10.0 mL with 70% methanol. All extractions were performed in triplicate. To ensure the reliability and stability of the analytical system, a quality control (QC) sample was prepared by pooling equal aliquots from each experimental extract.

### 2.3. UHPLC–Orbitrap–MS/MS Analysis

Metabolomic profiling was performed using a Vanquish UHPLC system coupled to an Orbitrap Exploris 120 mass spectrometer equipped with a heated electrospray ionization (HESI) source (Thermo Scientific, San Jose, CA, USA). Chromatographic separation was achieved on an Acquity UPLC HSS T3 column (2.1 mm × 100 mm, 1.8 µm; Waters, Milford, MA, USA) maintained at 30 °C, while the sample tray was kept at 4 °C. The injection volume was 2 µL, and the flow rate was set at 0.4 mL/min. Mobile phase A consisted of water containing 0.1% formic acid (*v*/*v*), and mobile phase B was acetonitrile containing 0.1% formic acid (*v*/*v*). The optimized gradient program was as follows: 0–3 min, 2–5% B; 3–8 min, 5–35% B; 8–17 min, 35–95% B; 17–18 min, 95% B; 18–19 min, 95–2% B; and 19–24 min, 2% B for column re-equilibration.

Mass spectrometry was conducted in positive electrospray ionization (ESI+) mode. The HESI source parameters were optimized to minimize in-source fragmentation as follows: spray voltage, 3.1 kV; ion transfer tube temperature, 320 °C; and vaporizer temperature, 400 °C. Nitrogen was used as the sheath, auxiliary, and purge gas at flow rates of 45, 20, and 5 arbitrary units, respectively. Data were acquired using a combination of full-scan and data-dependent MS2 (dd-MS2) modes. The MS1 resolution was set at 120,000 (FWHM at *m*/*z* 200) over a scan range of *m*/*z* 150–1500, while the MS2 resolution was 7500. For dd-MS2, the intensity threshold was 9 × 10^4^, and the normalized collision energy (NCE) was 35%. Dynamic exclusion was implemented with a repeat count of 1 and an exclusion duration of 10 s. System control and data acquisition were managed via Xcalibur software 4.1 (Thermo Scientific). To ensure system stability and data reproducibility, samples were analyzed in a randomized order. A QC sample, prepared by pooling equal aliquots from all experimental samples, was used to equilibrate the column (three initial injections) and was subsequently injected after every three samples throughout the analytical run [19]. Experimental blanks were injected at the beginning and end of the sequence to monitor and subtract background noise.

### 2.4. Data Processing and Multivariate Statistical Analysis

Raw LC-MS/MS data were processed using Compound Discoverer 3.4 (Thermo Fisher Scientific, USA) for peak detection, alignment, deconvolution, and normalization. The software generated a data matrix containing retention times (RT), mass-to-charge ratios (*m*/*z*), and normalized peak areas. Raw LC-MS/MS data were processed using Compound Discoverer 3.4 (Thermo Fisher Scientific, USA) for peak detection, alignment, deconvolution, and normalization. Based on previous reports [20], the normalized data matrix (relative standard deviation (RSD) < 30%) was imported into SIMCA software (v14.1, Umetrics, Umeå, Sweden) for multivariate statistical analysis. Principal Component Analysis (PCA) was first employed to visualize the overall metabolic grouping and monitor analytical stability. Subsequently, partial least squares discriminant analysis (PLS-DA), orthogonal partial least squares discriminant analysis (OPLS-DA) and hierarchical clustering analysis (HCA) were conducted to maximize the discrimination between *GE* tubers at different developmental stages. To identify significant differential metabolites, a stringent filtering strategy was implemented based on three integrated criteria: a Variable Importance in Projection (VIP) score > 1.0 derived from the OPLS-DA model, a *p*-value < 0.05 (adjusted using the Benjamini–Hochberg False Discovery Rate (FDR) correction), and a fold change (FC) of ≥1.5 or ≤0.67. Metabolite annotation was performed by matching accurate mass, isotopic patterns, and MS/MS fragmentation spectra against multiple electronic databases, including *mz*Cloud (https://www.mzcloud.org/, accessed on 21 March 2026), the Human Metabolome Database (HMDB, http://www.hmdb.ca/, accessed on 21 March 2026), and PubChem (https://pubchem.ncbi.nlm.nih.gov/, accessed on 21 March 2026). Identification was further validated through comparisons with published literature and, where available, by matching MS/MS spectra with authentic standards. A mass tolerance of 5 ppm was applied for molecular formula assignment. To visualize the metabolic patterns and accumulation trajectories across stages, a colorimetric heatmap combined with HCA was generated using MetaboAnalyst 5.0. The final metabolic pattern and KEGG visualization were achieved through hierarchical clustering analysis using MetaboAnalyst 5.0 (https://www.metaboanalyst.ca/), presented as a colorimetric heatmap.

### 2.5. Determination of Chemical Components

Chemical composition analysis was conducted utilizing well-established methods including the Folin–Ciocalteu method [21]. Briefly, the absorbance was recorded at 765 nm, and gallic acid was employed as the calibration standard; results were expressed as mg gallic acid equivalents. Total flavonoid content (TFC) was measured by the aluminum nitrate colorimetric method using rutin as the standard (λ = 510 nm) [22]. Total soluble sugars were quantified using the anthrone–sulfuric acid method (glucose standard, λ = 620 nm). Total free amino acids were determined by the ninhydrin method (glycine standard, λ = 570 nm) [23]. All measurements were performed in triplicate using a UV–Vis spectrophotometer (Shimadzu UV-2600, Kyoto, Japan), and results were expressed as mg/g of dry matter of *GE*. Detailed calibration curve parameters are provided in Table S1.

### 2.6. Determination of Antioxidant Activity

The antioxidant activities of the extracts were assessed using the DPPH• and ABTS•^+^ radical scavenging assays, as per the manufacturer’s instructions [24]. For the DPPH• assay, 20 μL of sample was mixed with 90 μL of DPPH• solution and incubated in the dark at 25 °C for 30 min. The absorbance was recorded at 517 nm. Radical scavenging activity (%) was calculated using the following formula:

$$DPPH\cdot radical\;scavenning\;rate\;(\%)\;=\;[\;(A_{0}-A_{1})\;/\;(A_{0}-A_{2})]\times 100\%\;$$

where *A*_0_ represents the absorbance of the solvent alone, *A*_1_ is the absorbance of the sample mixed with the radical solution, and A_2_ is the absorbance of the solvent alone. IC_50_ values were determined using nonlinear regression analysis. For the ABTS•^+^ assay, 20 μL of each sample solution at varying concentrations was mixed with 180 μL of ABTS•^+^ working solution and incubated at 25 °C for 6 min. Absorbance was measured at 405 nm, and the percentage inhibition was calculated using the same formula as described for the DPPH• assay.

### 2.7. α-Glucosidase and α-Amylase Inhibitory Assays

The inhibitory activities of the extracts on α-amylase and α-glucosidase were evaluated following previously reported methods with slight modifications [25]. For the α-glucosidase inhibition assay, 40 μL of each tea extract was mixed with 80 μL of α-glucosidase enzyme solution (1 U/mL in PBS, pH 6.8) and incubated in a 96-well plate at 37 °C for 10 min. Subsequently, 20 μL of pNPG (para-nitrophenyl-α-D-glucopyranoside) substrate was added to the mixture, and the reaction continued at 37 °C for an additional 5 min. The absorbance was then measured at 405 nm using a microplate reader (SpectraMax i3x, Molecular Devices, San Jose, CA, USA). The IC_50_ value was calculated through nonlinear regression analysis based on the logarithmic relationship between the inhibition percentage and the concentration of the sample extract. For the α-amylase inhibition assay, 100 μL of each *GE* methanol extract (or 1 mM acarbose as a positive control) was mixed with 100 μL of enzyme solution (1.1 U/mL in PBS, pH 6.8) and incubated at 37 °C for 15 min. After incubation, 50 μL of a 1% soluble starch solution in PBS was added, and the reaction continued at 37 °C for an additional 10 min. To terminate the reaction, DNS reagent was added, and the mixture was heated at 100 °C for 10 min. The absorbance was measured at 540 nm using a microplate reader (SpectraMax i3x, Molecular Devices, San Jose, CA). The α-amylase activity was then calculated following the previously established methodology [26].

### 2.8. Statistical Analysis

All experiments were conducted in triplicate, and results were expressed as mean ± standard error of the mean (SEM). Statistical significance among groups was evaluated using one-way ANOVA followed by post hoc tests (*p* < 0.05) using GraphPad Prism 8.0 (GraphPad Software, San Diego, CA, USA).

## 3. Results and Discussion

### 3.1. Significantly Different Chemical Profiles Among Three Growth Stages of GE

**Figure 1:**
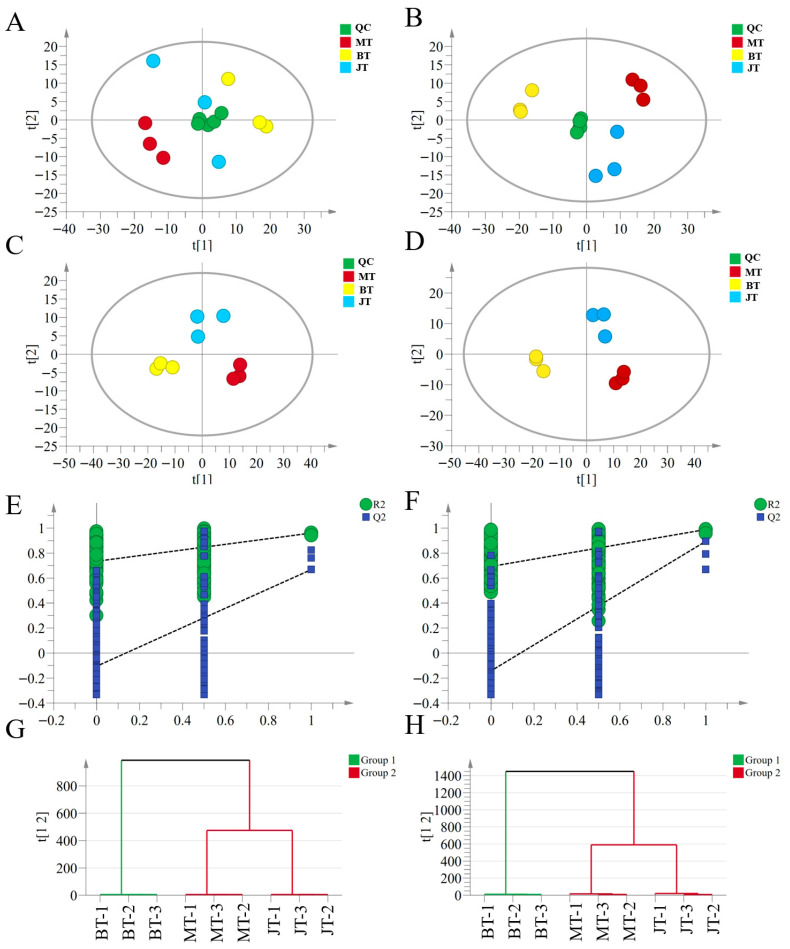
Multivariate statistical analysis of metabolites across three growth stages of *GE*. (**A**,**B**) PCA score plots obtained in positive and negative ionization modes. (**C**,**D**) PLS-DA score plots of permutation tests (*n* = 200) in positive (R^2^X = 0.806; R^2^Y = 0.985; Q^2^ = 0.892) and negative (R^2^X = 0.809; R^2^Y = 0.985; Q^2^ = 0.892) ionization modes, respectively. (**E**,**F**) Permutation test results for validation of the PLS-DA models in positive and negative ionization modes. (**G**,**H**) HCA of samples from the three growth stages based on metabolite profiles acquired in positive and negative ionization modes, respectively, further illustrating distinct metabolic clustering patterns among stages.

To elucidate the metabolic differences in *GE* across distinct developmental stages, an untargeted metabolomics strategy based on UHPLC-Orbitrap-MS/MS was applied to analyze samples collected from three growth stages (T). As previously documented, untargeted metabolomics approaches have shown significant potential for the comprehensive profiling of metabolites in plants [12,27]. These methods have been widely applied to analyze metabolic profile variations across different growth stages or edible plant parts [28,29]. A total of 3641 ion features in positive ion mode and 2725 ion features in negative ion mode were detected (Figure S1), respectively. In the positive ion mode, MT, BT, and JT samples were clearly distributed in distinct quadrants with minimal overlap (Figure 1A), indicating pronounced metabolic differences. A similar separation pattern was observed in the negative ion mode (Figure 1B). Moreover, QC samples clustered tightly in both ion modes, demonstrating the stability and reliability of the analytical platform. These findings suggest that the three growth stages of *GE* possess significantly different chemical profiles. Given that PLS-DA offers enhanced discrimination over PCA in assessing group variation, supervised PLS-DA was performed to further investigate stage-dependent differences [30]. The PLS-DA score plots showed clear separation among MT, BT, and JT in both positive and negative ion modes (Figure 1C,D), further confirming distinct chemical compositions across growth stages. The model parameters demonstrated excellent explanatory and predictive performance. Specifically, the R^2^X, R^2^Y, and Q^2^ values were 0.806, 0.985, and 0.892 in the positive ion mode, and 0.809, 0.973, and 0.951 in the negative ion mode, respectively (Figure 1E,F). These results indicate that the models were successfully established with strong goodness-of-fit and predictive ability. Furthermore, permutation tests validated the robustness of the PLS-DA models, confirming the absence of overfitting in both ion modes (Figure 1G-H). Hierarchical cluster analysis (HCA) was subsequently applied to further explore the similarities among samples. The results showed that MT and JT samples within the growth stage clustered together first, while the BT samples each exhibited independent clusters, and showed distinct profiles from the other grades. Collectively, these results provide strong evidence that developmental progression is accompanied by distinguished chemical profiles.

### 3.2. Characteristic Metabolites Among Three Growth Stages of GE

**Figure 2:**
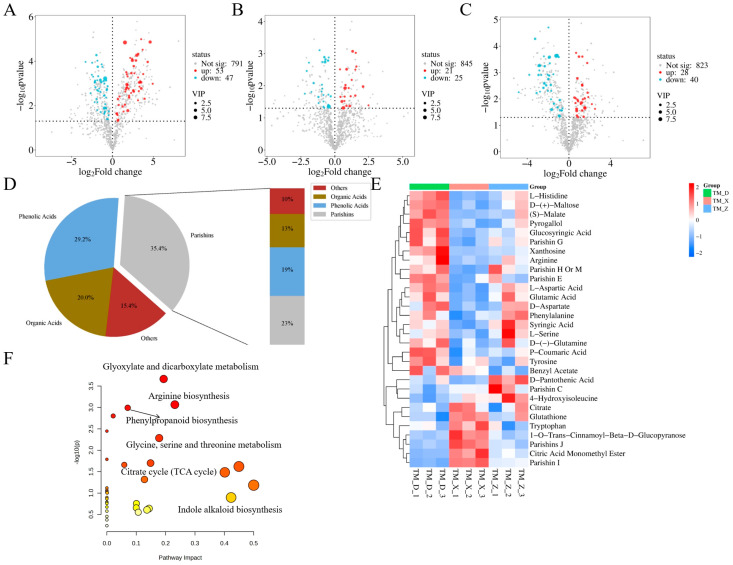
Differential metabolite analysis and metabolic pathway enrichment among MT, BT, and JT groups. (**A**–**C**) Volcano plots illustrating the differential metabolites between MT vs. BT (**A**), MT vs. JT (**B**), and BT vs. JT (**C**). Red dots represent significantly up-regulated metabolites; blue dots represent down-regulated metabolites. (**D**) Chemical classification and proportion of the identified differential metabolites. (**E**) Hierarchical clustering heatmap of key differential metabolites across MT, BT, and JT groups. The color scale from blue to red represents relative abundance from low to high. (**F**) KEGG pathway enrichment analysis of the differential metabolites.

OPLS-DA has been reported to provide superior discriminatory performance compared with PCA for evaluating intergroup variation [31]. Therefore, OPLS-DA was further applied to assess potential differences among the sample groups. As shown in Figure S2, samples from each growth stage of *GE* were clearly separated from one another, and the permutation tests validated the robustness of the models (Figure S3). According to the previously described method [27], differential metabolites were screened using the criteria of VIP > 1.0, FDR-adjusted *p*< 0.05, and FC ≥ 1.5 or ≤0.67. Volcano plot analysis (Figure 2A–C) revealed pronounced metabolic alterations during development. A total of 100 differential metabolites were identified between BT and MT (53 up-regulated and 47 down-regulated). In addition, 46 and 68 differential metabolites were detected in the MT vs. JT and BT vs. JT comparisons, respectively (VIP > 1.0, *p* < 0.05), indicating dynamic metabolic reprogramming across growth stages. The identified metabolites were structurally characterized by comparing their retention time (Rt), *m*/*z* values, and MS/MS fragmentation patterns with reference standards, published literature, and databases, as summarized in Table 1. Among them, 31 key differential metabolites were selected, including 11 amino acids, seven parishins, six phenolic acids, four organic acids, and three other compounds (Figure 2E). These results indicate that amino acids, parishins, and phenolic acids represent the predominant metabolic signatures associated with *GE* maturation. Although this approach enables the broad detection of metabolites and facilitates the discovery of potential biomarkers, the structural annotation of most certain compounds still relies on database matching and MS/MS fragmentation patterns. Therefore, further confirmation using authentic standards or complementary analytical techniques would strengthen the reliability of metabolite identification.

A heatmap illustrating the relative abundance of these 31 metabolites clearly visualized the distinct metabolic profiles among the three developmental stages (Figure 2E). Furthermore, KEGG pathway enrichment analysis (Figure 2F) demonstrated that the differential metabolites were mainly involved in glyoxylate and dicarboxylate metabolism, arginine biosynthesis, and phenylpropanoid biosynthesis (pathway impact > 0.1). Notably, amino acid metabolism plays a crucial role in plant development and in the biosynthesis of secondary metabolites [32,33]. Collectively, these findings suggest a developmental metabolic shift from primary nitrogen metabolism toward enhanced secondary biosynthesis of gastrodin or phenolic compounds during *GE* tuber maturation.

#### 3.2.1. Amino Acids

**Figure 3:**
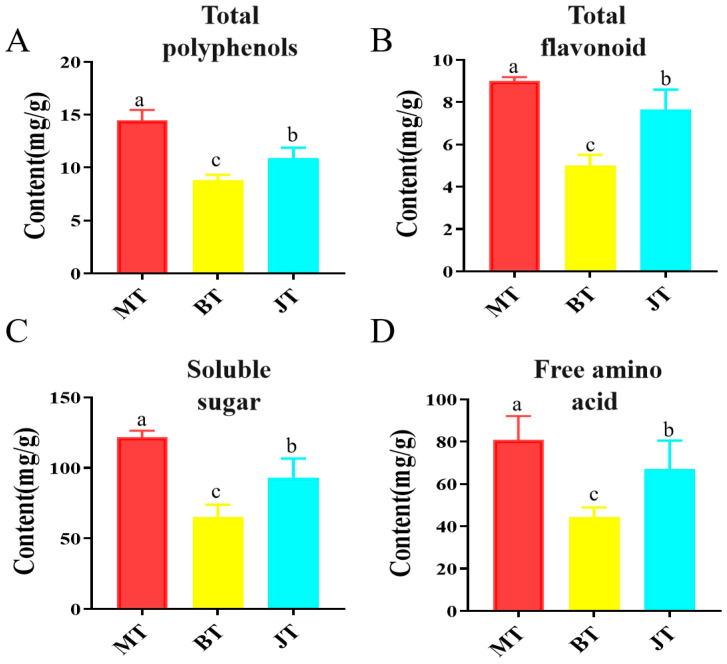
Quantitative comparison of four bioactive compounds across three growth stages of *GE*. (**A**) Total polyphenol content; (**B**) total flavonoid content; (**C**) soluble sugar content; (**D**) free amino acid content. Values are means and SEM (*n* = 3). Columns with different letters are significantly different (*p* < 0.05).

Amino acids are crucial compounds in plant metabolism, contributing significantly to the taste and nutritional quality of plants [34,35]. Comparative metabolomic analysis demonstrated significant variations in the levels of several amino acids among the three growth stages. As shown in Table 1 and Figure 3, tryptophan, L-aspartic acid, D-aspartate, and glutamic acid exhibited pronounced stage-dependent differences. Specifically, tryptophan and glutamic acid accumulated at significantly higher levels in BT than in MT, whereas D-aspartate was more abundant in JT. These distinct accumulation patterns suggest differential metabolic priorities across developmental stages. From a metabolic perspective, glutamic acid is a key intermediate in nitrogen metabolism and participates in transamination reactions that regulate amino acid biosynthesis [36]. Aspartic acid is also involved in the aspartate family amino acid pathway, which contributes to plant growth and metabolic regulation [37]. In addition, tryptophan is an important precursor for the synthesis of various secondary metabolites [38]. Therefore, the observed variations in amino acid levels across developmental stages may reflect shifts in nitrogen metabolism and biosynthetic activity during tuber development.

#### 3.2.2. Parishins

Parishins, a unique group of esterified derivatives formed by the condensation of gastrodin and citric acid, are recognized as key bioactive constituents of *GE* and contribute substantially to its neuroprotective and pharmacological activities [39,40,41]. Previous studies have demonstrated that parishins isolated from *GE* roots exert cardioprotective effects (Wang et al.) [41], while Liu et al. reported that parishin alleviates DSS-induced colitis and anxiety-like behavior in mice by modulating intestinal barrier function and the microbiota–gut–brain axis [42]. In addition, gastrodin and parishin C have been implicated in promoting PI3K/Akt-mediated neurogenesis via targeting EGFR in neural stem/progenitor cells, suggesting their potential relevance in the prevention and treatment of neurological disorders [43]. In the present study, the accumulation patterns of parishin derivatives and their primary precursor, gastrodin, exhibited pronounced stage-dependent variations across MT, BT, and JT. Parishin E was the most abundant compound within this class, reaching its highest level at the early MT stage, together with parishin G. As development progressed to BT, a clear metabolic transition was observed, characterized by maximal accumulation of parishin and parishin J. Moreover, gastrodin and parishin C displayed a continuous increasing trend, with peak concentrations detected at the JT stage. These dynamic fluctuations indicate an active metabolic flux involving the biosynthesis, interconversion, and potential enzymatic hydrolysis of parishin analogs during tuber development.

#### 3.2.3. Phenolic Acids and Their Derivatives

The *GE* was found to be rich in phenolic acids, which are well known for their antioxidant properties [44]. Phenolic compounds, such as p-coumaric acid, syringic acid, and pyrogallol, were among the differentially accumulated metabolites, exhibiting distinct patterns across the growth stages (Figure 3). Specifically, p-coumaric acid and syringic acid concentrations were significantly higher in JT, while pyrogallol levels were notably elevated in BT. In a similar study, Li et al. reported an increase in quinic acid content in Yuexi Cuilan tea with leaf maturity, with the youngest leaves containing the lowest levels, a result that aligns with our findings [45]. These phenolic compounds are mainly produced through the phenylpropanoid metabolic pathway, which plays an important role in plant defense and stress responses [46]. The increased accumulation of certain phenolic acids in the mature stage may be associated with enhanced antioxidant capacity and metabolic adaptation during tuber development. A similar developmental trend has been reported in tea plants, where quinic acid content increased with leaf maturity [47], which is consistent with our observations.

#### 3.2.4. Organic Acids

Organic acids are essential for various physiological functions in plants, including regulation of pH, nutrient absorption, and flavor enhancement [48]. In this study, significant variations were found in the levels of organic acids such as citrate, (S)-malate, and citric acid monomethyl ester across the different growth stages (Figure 3). Notably, the highest levels of citrate and (S)-malate were found in JT, suggesting that the metabolic processes associated with energy production and stress responses are more pronounced in the later stages of growth.

#### 3.2.5. Other Metabolites

Additionally, several other metabolites, including Xanthosine, Glutathione and Maltose, were identified as differentially abundant across the three growth stages. Xanthosine and Maltose showed higher concentrations in MT, while Glutathione was more abundant in JT. These findings suggest that the accumulation of certain secondary metabolites, such as those involved in aroma and flavor production, also varies depending on the growth stage.

### 3.3. Changes in Active Components During Three Growth Stages of GE

To further validate the metabolic shifts identified via untargeted metabolomics, the primary and secondary bioactive components of G. elata tubers at different growth stages were quantitatively assessed. As shown in Figure 3A,B, the secondary metabolite profiles—specifically total phenolics and total flavonoids—exhibited significant fluctuations during tuber development. The TPC in the MT reached 14.50 mg/g, which was substantially higher than that observed in the immature (BT, 8.78 mg/g) and mature stages (JM, 10.88 mg/g). A similar trend was observed for total flavonoid content (TFC), where MT (8.99 mg/g) outperformed BT (5.01 mg/g) and JT (6.66 mg/g). These results indicate that the early developmental stage of G. elata is characterized by a higher density of bioactive secondary metabolites, aligning with our previous UHPLC-Orbitrap-MS/MS findings. Notably, this pattern of higher phenolic accumulation in earlier tissues is consistent with research by Ma et al., who observed analogous variations across different maturity stages in lotus leaf extracts [49], suggesting a common biological strategy where young tissues may prioritize chemical defense through secondary metabolite enrichment.

Soluble sugars and free amino acids are equally vital, serving as both nutritional constituents and precursors for various metabolic pathways in *GE* [50]. Quantitative assessments revealed that these primary metabolites followed a “U-shaped” or “declining-then-recovering” trajectory across the three stages (Figure 3C,D). The total sugar content was highest in the MT stage (121.84 mg/g), significantly decreased during the rapid expansion phase in BT (65.10 mg/g), and partially recovered in the mature JT stage (89.66 mg/g). Similarly, total amino acids were most abundant in MT (80.81 mg/g), followed by a sharp decline in BT (44.47 mg/g) and a subsequent increase in JT (67.02 mg/g). The significant reduction in these components in the intermediate BT stage suggests high metabolic consumption during the peak growth phase, where energy and nitrogen sources are rapidly utilized for biomass accumulation. Amino acids and their derivatives are instrumental in shaping the intricate flavor profile and medicinal efficacy of G. elata. This study demonstrates that while the absolute concentration of these nutrients is highest in MT, the JM achieves a secondary equilibrium of chemical richness. These discoveries deepen our comprehension of the physiological transformation of *GE* during maturation.

### 3.4. Change in Antioxidant Activity, α-Glucosidase and α-Amylase Inhibitory Activity of GE During Three Growth Stages

ABTS•^+^ and DPPH• radical scavenging assays are well-established and widely employed antioxidant methods, serving as indicators of a sample’s capacity to donate hydrogen atoms. In this study, the antioxidant activities of *GE* extracts from three developmental stages were assessed by determining their IC50 values against ABTS•^+^ and DPPH• radicals. As summarized in Table 2, all *GE* extracts exhibited dose-dependent scavenging capacities against both DPPH and ABTS radicals. The IC_50_ values (the concentration required to achieve 50% radical inhibition) for DPPH scavenging in MT, BT, and JT were determined to be 32.35 2.02, 51.25 2.96, and 42.28 + 3.44 mg/mL, respectively. Similarly, the IC50 values for ABTS radical scavenging followed a comparable trend, with MT (32.31 + 0.81 mg/mL) demonstrating the most potent activity, followed by JT (36.58 + 1.93 mg/mL) and BT (38.93 I 0.81 mg/mL). These results unequivocally demonstrate that *GE* tubers at various growth stages possess positive antioxidant capacities. Notably, the MT exhibited a more pronounced free radical scavenging ability compared to the BT and JM stages. This superior performance in MT aligns with our prior metabolic profiling, which revealed a higher concentration of total phenolics and flavonoids during the early growth stage, suggesting that these bioactive constituents are the primary contributors to the observed antioxidant efficacy.

α-Glucosidase and α-amylase are key enzymes involved in carbohydrate digestion and glucose absorption. Inhibition of these enzymes is an established strategy for managing postprandial hyperglycemia. Our in vitro experiments demonstrated that water extracts of three various *GE* extracts inhibited the activity of α-glucosidase and α-amylase. As shown in Table 2, all three extracts exhibited inhibitory effects on α-glucosidase activity. The IC_50_ values followed the order MT < JT < BT, indicating that MT possessed the strongest inhibitory capacity, whereas BT showed the weakest effect. A similar trend was observed for α-amylase inhibition, with MT demonstrating the lowest IC_50_ value and thus the highest inhibitory activity. The superior inhibitory effects of MT may be attributed to its higher contents of polyphenols, flavonoids, and certain parishin derivatives, which have been reported to contribute to carbohydrate-hydrolyzing enzyme inhibition [51]. In contrast, the relatively lower activity observed in BT is consistent with its lower levels of these bioactive constituents.

### 3.5. Correlation Analysis Between Active Components and Bioactivities in Three Growth Stages of GE

**Figure 4:**
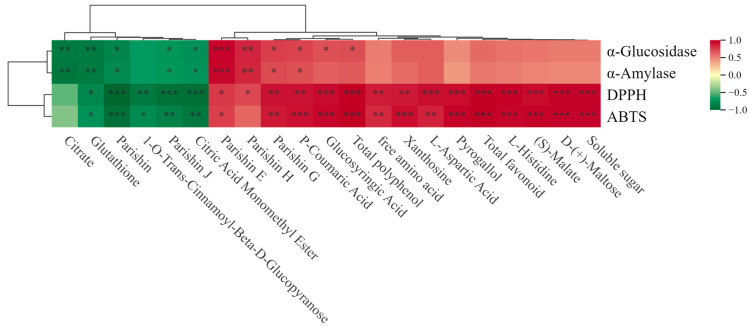
Correlation heat map analysis between active components and bioactivities during the three growth stages of *GE*.* *p* < 0.05, ** *p* < 0.01, *** *p* < 0.001.

As shown in Figure 4, Pearson correlation analysis revealed significant associations between major metabolites and the bioactivities of *GE* at different developmental stages. Notably, antioxidant activities exhibited strong correlations with several phenolic-related components. In particular, DPPH• radical scavenging capacity exhibited a very strong correlation with total flavonoid content (R^2^ > 0.95), while ABTS•^+^ scavenging activity was strongly correlated with total phenolic content (R^2^ > 0.95). In addition, pyrogallol, L-histidine, and (S)-malate showed significant correlations with antioxidant indices (|r| > 0.8, *p* < 0.001). For hypoglycemic-related activities, α-amylase inhibition was strongly correlated with total flavonoid content (R^2^ > 0.95), whereas α-glucosidase inhibition was more closely associated with total phenolic content (R^2^ > 0.90). Notably, Parishin E, Parishin G, and Parishin H displayed significant positive correlations with both enzyme inhibitory activities (*p* < 0.05 or *p* < 0.01), indicating that these characteristic phenolic glycosides may be key contributors to carbohydrate-digesting enzyme inhibition. In contrast, organic acids (e.g., citrate) and glutathione showed weak or negative associations with α-amylase and α-glucosidase inhibition (R^2^ < 0.5), suggesting a limited contribution to hypoglycemic activity. Correlation analysis indicated that phenolic- and flavonoid-related metabolites, particularly parishin derivatives (e.g., Parishin E, Parishin G, and Parishin H), showed strong associations with antioxidant capacity and α-glucosidase/α-amylase inhibitory activities. These results suggest that parishin derivatives and phenolic compounds may represent the major bioactive constituents contributing to the pharmacological effects of G. elata and therefore may serve as promising targets for further pharmacological investigation. However, these correlations should be interpreted cautiously, as they represent preliminary associations rather than direct causal relationships. Further studies involving larger sample sizes targeted, metabolite validation, mechanistic investigations, and in vivo experiments will be necessary to confirm the pharmacological roles of these metabolites.

## 4. Conclusions

In this study, the effects of growth stages on the chemical composition and bioactivities of *GE* were systematically evaluated. Metabolomic analysis revealed clear stage-dependent discrimination in metabolic profiles, and a total of 31 differential metabolites were identified, including parishin derivatives, phenolics, amino acids, and organic acids. The results demonstrated that developmental stage markedly influences the accumulation of total phenolics, total flavonoids, specific parishin derivatives, and their associated bioactivities. Tubers at the early developmental stage (MT) exhibited the highest TPC and TFC, together with the strongest antioxidant capacity and carbohydrate-hydrolyzing enzyme inhibitory activities. Most parishin derivatives (e.g., parishin E and G) and flavonoids showed a pronounced decline during tuber development, whereas gastrodin and parishin C displayed the opposite trend, reaching peak levels at the mature stage (JT). Antioxidant activity and α-glucosidase/α-amylase inhibitory effects were positively correlated with TPC, TFC, and representative parishin analogs (parishin E, G, and H), highlighting their major contributions to *GE* bioactivity.

Nevertheless, this study is limited to in vitro activity evaluations, and further in vivo investigations are required to substantiate the observed biological effects. Collectively, these findings provide a scientific basis for stage-specific grading and targeted utilization of *GE* tubers, and offer practical guidance for optimizing harvest timing to maximize the functional and medicinal value of *GE*-derived products and functional foods.

## Figures and Tables

**Table 1:** The identification of differentiated metabolites among three growth stages of *GE* samples based on UPLC-Q-orbitrap-MS/MS.

No.	Compound Name	Formula	RT [min]	Reference Ion	Adduct *m*/*z*	Error (ppm)	Fragments	FC (BT/MT)	FC (JT/MT)
	**Amino Acid**								
1	Tryptophan	C_11_H_12_N_2_O_2_	2.66	[M + H]+1	205.09711	−0.22	188, 146, 118	1.62	0.83
2	L-Aspartic Acid	C_4_H_7_NO_4_	1.15	[M + H]+1	134.04482	0.29	134	0.47	0.84
3	D-aspartate	C_4_H_7_NO_4_	1.08	[M-H]−1	132.02918	−4.89	132, 88	0.38	0.83
4	D-(-)-Glutamine	C_5_H_10_N_2_O_3_	1.16	[M + H]+1	147.07638	−0.24	146	0.63	0.87
5	Glutamic Acid	C_5_H_9_NO_4_	1.15	[M + H]+1	148.06036	−0.16	130, 84	0.57	0.96
6	4-Hydroxyisoleucine	C_6_H_13_NO_3_	1.16	[M + H]+1	148.09671	−0.54	148	1.23	1.43
7	L-Serine	C_3_H_7_NO_3_	1.16	[M + H]+1	106.05015	2.66	106	0.67	1.18
8	L-Histidine	C_6_H_9_N_3_O_2_	1.14	[M + H]+1	156.07678	0.15	156, 111, 95	0.22	0.60
9	Phenylalanine	C_9_H_11_NO_2_	1.75	[M + H]+1	166.08627	0.11	120, 93	0.72	1.04
10	Arginine	C_6_H_14_N_4_O_2_	1.13	[M + H]+1	175.11894	−0.09	175, 116, 70, 60	0.22	0.55
11	Tyrosine	C_8_H_8_O_3_	1.45	[M + H]+1	182.08117	0.02	166, 136, 123	0.83	0.80
	**Parishins**								
12	Parishin G *	C_19_H_24_O_13_	1.59	[M-H]−1	459.11487	1	173, 111	0.42	0.71
13	Parishin E *	C_19_H_24_O_13_	2.13	[M-H]−1	459.11454	0.31	173, 111	0.66	0.76
14	Parishins J	C_20_H_26_O_13_	3.28	[M-H]−1	473.13028	−0.05	169, 111, 73	3.64	1.34
15	Parishin H	C_33_H_42_O_20_	3.53	[M-H]−1	757.22064	−0.47	423, 161, 111	0.40	0.93
16	Parishin I	C_38_H_50_O_24_	2.91	[M-H]−1	889.26329	−3.24	423, 161, 111	6.52	3.12
17	Parishin C *	C_32_H_40_O_19_	3.45	[M + NH4]+1	746.24939	−1.18	697, 269, 107	1.28	1.70
18	Gastrodin *	C_13_H_18_O_7_	2.35	[M-H]-	285.098	0.21	285, 267, 241, 123, 93	1.30	1.62
	**Phenolic Acids**								
19	Glucosyringic Acid	C_15_H_20_O_10_	1.70	[M-H]−1	359.09864	0.72	359, 197, 123	0.45	0.62
20	Pyrogallol	C_6_H_6_O_3_	1.19	[M + H]+1	127.03909	0.92	127, 109, 81	0.61	0.72
21	1-O-Trans-Cinnamoyl-Beta-D-Glucopyranose	C_15_H_18_O_7_	5.15	[M + Na]+1	333.09422	−0.8	333, 185	3.93	1.21
22	Syringic Acid	C_9_H_10_O_5_	1.70	[M-H]−1	197.04494	−3.04	197, 182, 160, 123	0.56	1.21
23	Hydrocinnamic acid	C_9_H_10_O_2_	1.51	[M + H]+1	151.07538	0.13	151, 136, 107, 91	0.88	0.48
24	P-Coumaric Acid	C_9_H_11_NO_3_	1.20	[M + NH4]+1	182.08116	−0.02	136	0.73	0.81
	**Organic Acid**								
25	D-Pantothenic Acid	C_9_H_17_NO_5_	1.62	[M-H]−1	218.10298	−1.88	218, 146, 88	0.84	1.39
26	(S)-Malate	C_4_H_6_O_5_	1.14	[M + FA-H]−1	133.01318	−1.41	133, 115, 71	0.53	0.68
27	Citrate	C_6_H_8_O_7_	1.13	[M-H]−1	191.01897	−3.93	111	1.40	1.09
28	Citric Acid Monomethyl Ester	C_7_H_10_O_7_	1.51	[M-H]−1	205.03482	−2.71	173, 111	4.15	1.95
	**Others**								
29	Xanthosine	C_10_H_12_N_4_O_6_	2.19	[M-H]−1	282.06192	−3.75	282, 167, 123	0.07	0.24
30	Glutathione	C_10_H_17_N_3_O_6_S	1.45	[M + H]+1	308.09084	−0.78	179, 162, 149, 84, 76	1.58	1.26
31	Melibiose	C_12_H_22_O_11_	1.13	[M + K]+1	381.079	−0.99	381	0.53	0.74

Note: *: Confirmation in comparison with authentic standards; RT: retention time.

**Table 2:** IC50 values of *GE* from three growth stages for antioxidant and enzyme inhibitory activities.

Sample	MT	BJ	JT
DPPH IC_50_ (mg/mL)	32.35 ± 2.02 ^a^	51.25 ± 2.90 ^c^	42.28 ± 3.34 ^b^
ABTS IC_50_ (mg/mL)	32.31 ± 0.82 ^a^	38.93 ± 0.81 ^c^	36.58 ± 1.92 ^b^
α-Glucosidase IC_50_ (mg/mL)	2.38 ± 0.13 ^a^	2.38 ± 0.13 ^c^	2.63 ± 0.36 ^b^
α-AmylaseIC_50_(mg/mL)	5.25 ± 0.28 ^a^	5.25 ± 0.28 ^c^	5.80 ± 0.80 ^b^

Note: Different letters within columns denote statistically significant differences between groups (*p* < 0.05).

## Data Availability

The original contributions presented in this study are included in the article. Further inquiries can be directed to the corresponding author(s).

## Supplementary Materials

The following supporting information can be downloaded at https://www.mdpi.com/article/10.3390/metabo16040223/s1: Figure S1: Total ion current based on a UPLC-Q-orbitrap-MS/MS chromatogram. (A) Positive ion mode; (B) negative ion mode. Figure S2. OPLS-DA score plots of *GE* samples at three growth stages based on metabolomic profiling. Figure S3. The OPLS-DA model validation results for positive and negative ion modes. Table S1. Calibration curves and correlation coefficients for quantitative determination of major bioactive compounds. Table S2. The identification of differentiated metabolites’ information among three growth stages of *GE* samples.
