# Supplementary material for: Comparative Metabolomics Analysis of Gastrodia elata Blume Different Growth Stages: Insights into Chemical Composition and Bioactivities
Source: Metabolites. 2026 Mar 30;16(4):223. doi: 10.3390/metabo16040223 (PMC13117156; doi:10.3390/metabo16040223)
Supplement: Supplementary file 1 [file metabolites-16-00223-s001.zip › metabolites-4214557-supplementary.pdf]

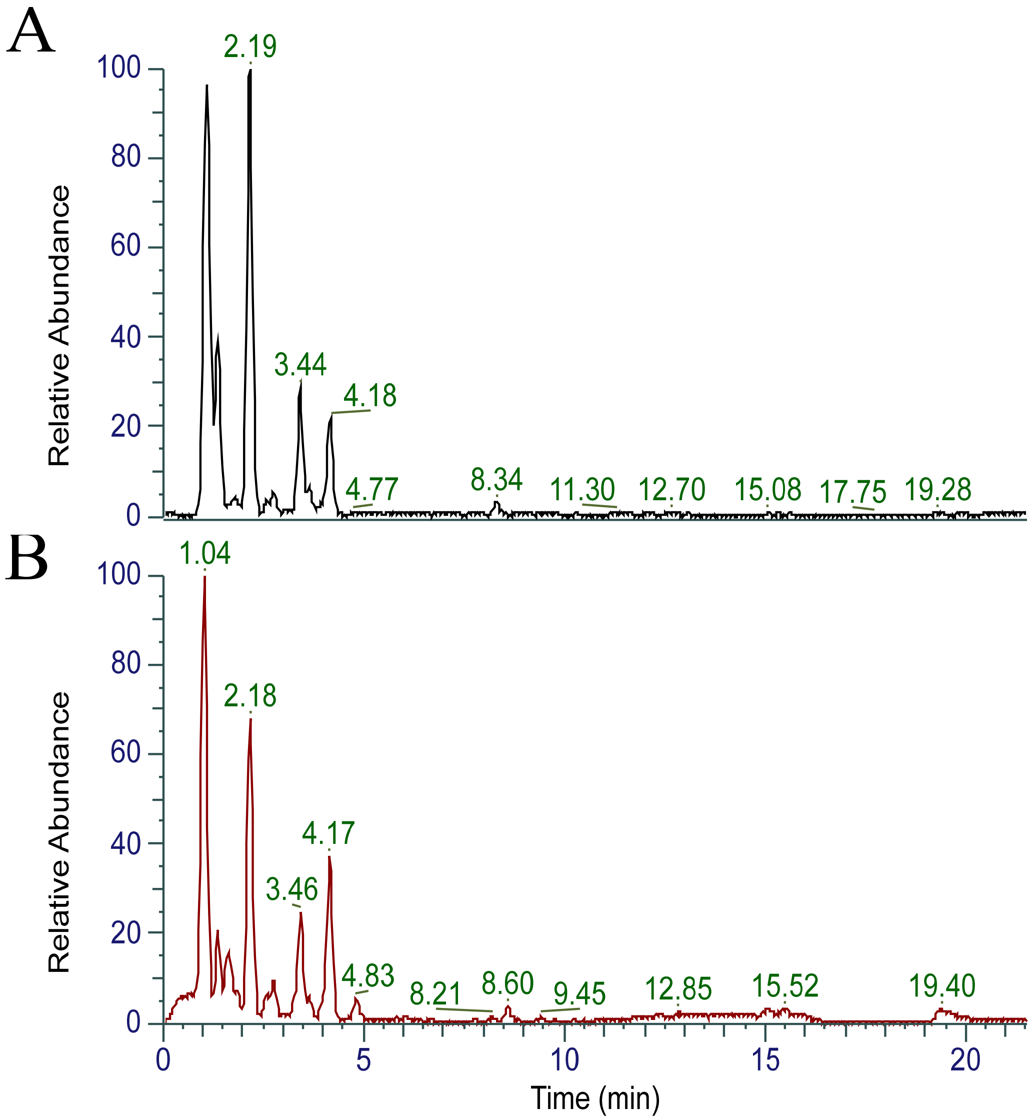

Figure S1. Total ion current based on a UPLC-Q-orbitrap-MS/MS chromatogram. (A) Positive ions mode; (B) negative ions mode.

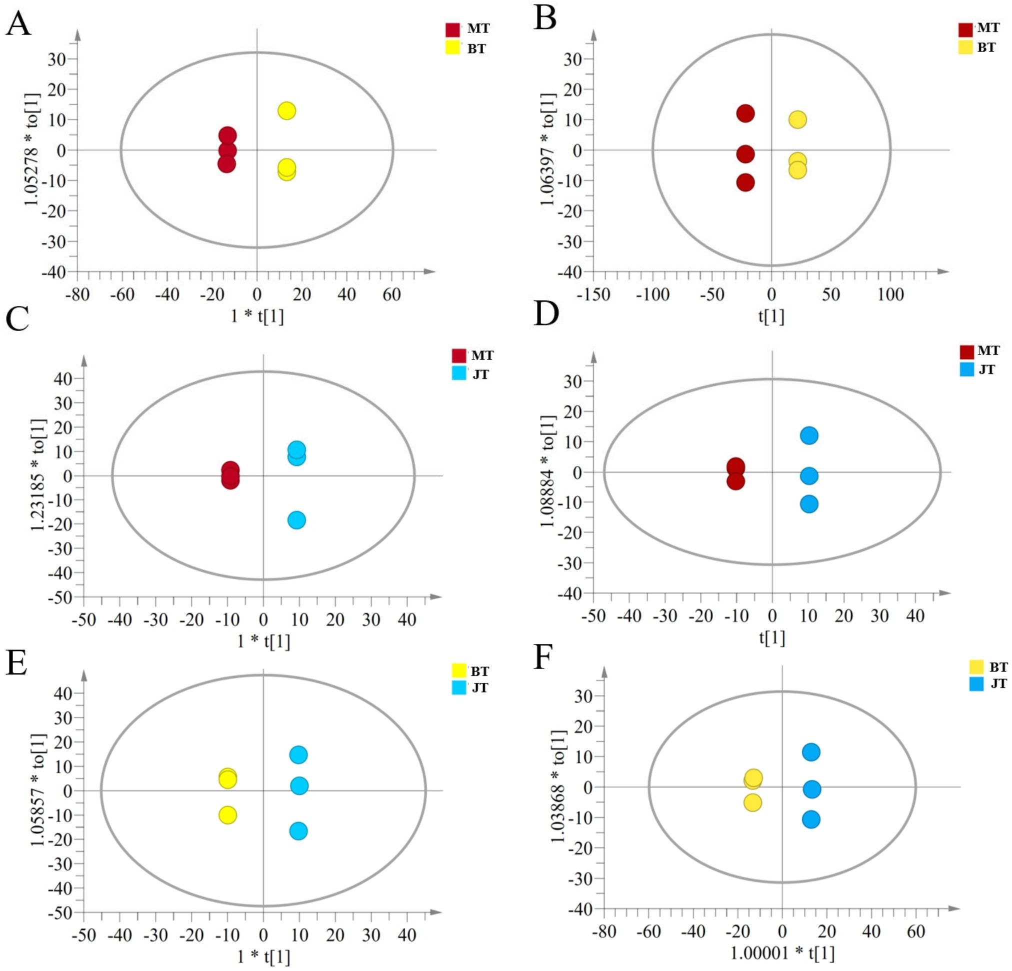

Figure S2. OPLS-DA score plots of GE samples at three growth stages based on metabolomic profiling. Left panels represent data acquired in negative ion mode (ESI−), and right panels represent data acquired in positive ion mode (ESI+). (A, B) MT vs. BT; (C, D) MT vs. JT; (E, F) BT vs. JT.

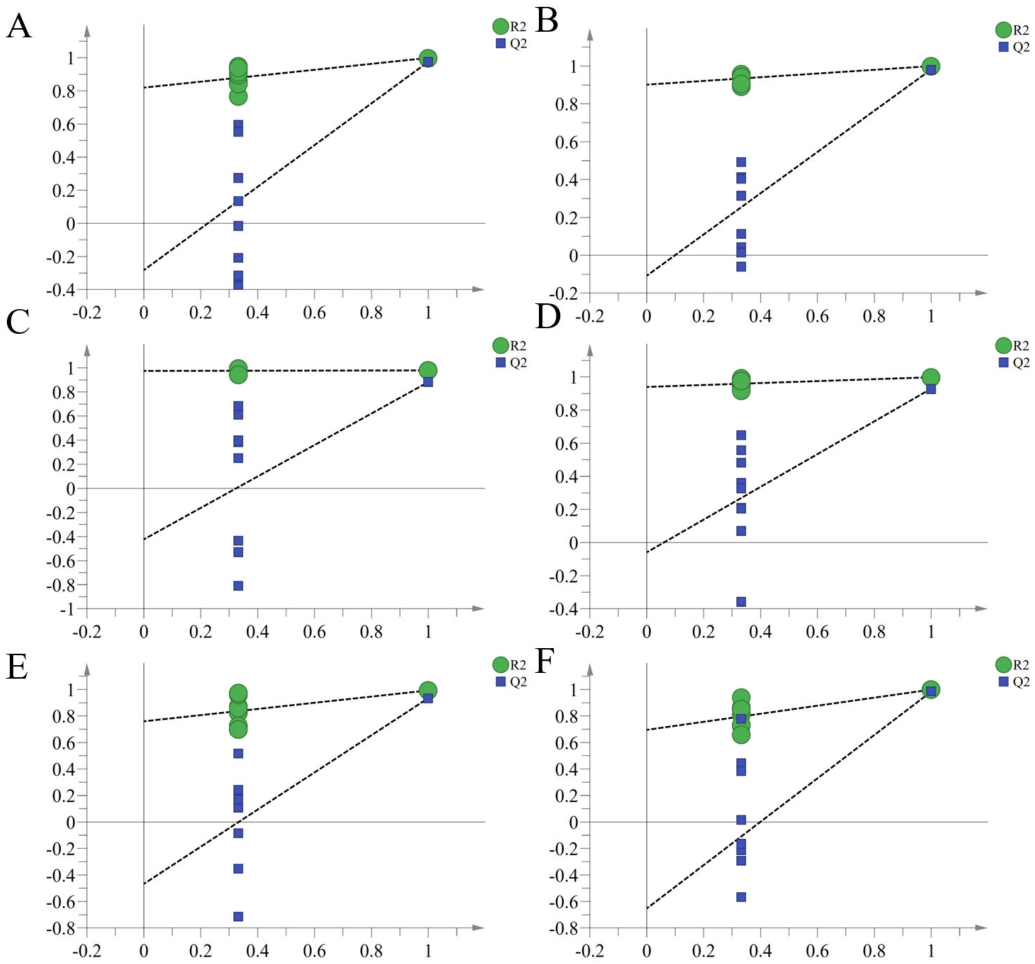

Figure S3. The OPLS-DA model validation results for positive and negative ion modes. Left panels represent data acquired in negative ion mode (ESI−), and right panels represent data acquired in positive ion mode (ESI+). (A, B) MT vs. BT; (C, D) MT vs. JT; (E, F) BT vs. JT.

Table S1. Calibration curves and correlation coefficients for quantitative determination of major bioactive compounds.

Analyte	Reference compound	Regression equation	R^2
Total flavonoids	Rutin	$y = 37.78x + 0.05$	0.999
Total phenolics	Gallic acid	$y = 22.67x + 0.05$	0.999
Total sugar	Glucose	$y = 2.60x + 0.08$	0.998
Total amino acids	Glycine	$y = 3.78x + 0.05$	0.998

Table S2. The identification of differentiated metabolites information among three growth stages o*f GE* samples.

NO.	Name	Formula	RT [min]	VIP	FDR	FC	
		MT vs BT					
1	D-Aspartate	$C_4H_7NO_4$	1.08	2.48	0.0383	0.38	Amino Acid
2	D-(+)-Maltose	$C_{12}H_{22}O_{11}$	1.13	9.90	0.0006	0.53	Others
3	Arginine	$C_6H_{14}N_4O_2$	1.13	4.64	0.0455	0.22	Amino Acid
4	(S)-Malate	$C_4H_6O_5$	1.14	4.83	0.0007	0.53	Organic Acid
5	L-Histidine	$C_6H_9N_3O_2$	1.14	2.28	0.0009	0.22	Amino Acid
6	L-Aspartic Acid	$C_4H_7NO_4$	1.15	2.11	0.0008	0.47	Amino Acid
7	Glutamic Acid	$C_5H_9NO_4$	1.15	3.16	0.0132	0.57	Amino Acid
8	D-(-)-Glutamine	$C_5H_{10}N_2O_3$	1.16	1.50	0.0405	0.63	Amino Acid
9	Pyrogallol	$C_6H_6O_3$	1.19	1.45	0.0020	0.61	Phenolic Acids
10	P-Coumaric Acid	$C_9H_{11}NO_3$	1.20	1.59	0.0182	0.73	Phenolic Acids
11	Gastrodin	$C_{13}H_{18}O_7$	2.35	2.30	0.0051	1.58	Parishins
12	Citric Acid Monomethyl Ester	$C_7H_{10}O_7$	1.51	4.20	0.0016	4.15	Organic Acids
13	Parishin G	$C_{19}H_{24}O_{13}$	1.59	1.72	0.0129	0.42	Parishins
14	Glucosyringic Acid	$C_{15}H_{20}O_{10}$	1.70	1.89	0.0080	0.45	Phenolic Acids
15	Parishin E	$C_{19}H_{24}O_{13}$	2.13	11.52	0.0090	0.66	Parishins

NO.	Name	Formula	RT [min]	VIP	FDR	FC	
16	Xanthosine	$C_{10}H_{12}N_4O_6$	2.19	1.50	0.0085	0.07	Others
17	Parishin I	$C_{38}H_{50}O_{24}$	2.91	4.37	0.0000	6.52	Parishins
18	Parishins J	$C_{20}H_{26}O_{13}$	3.28	1.53	0.0003	3.64	Parishins
19	Parishin H	$C_{33}H_{42}O_{20}$	3.53	1.66	0.0047	0.40	Parishins
20	Parishin C	$C_{32}H_{40}O_{19}$	3.75	1.59	0.0419	1.58	Parishins
21	1-O-Trans-Cinnamoyl-Beta-D-Glucopyranose	$C_{15}H_{18}O_7$	5.15	1.45	0.0017	3.93	Phenolic Acids
		JT vs MT					
1	D-(+)-Maltose	$C_{12}H_{22}O_{11}$	1.13	9.33	0.0447	0.74	Others
2	(S)-Malate	$C_4H_6O_5$	1.14	4.87	0.0497	0.68	Organic Acids
3	L-Histidine	$C_6H_9N_3O_2$	1.14	2.04	0.0477	0.60	Amino Acid
4	4-Hydroxyisoleucine	$C_6H_{13}NO_3$	1.16	0.99	0.0185	1.53	Amino Acid
5	Pyrogallol	$C_6H_6O_3$	1.19	1.55	0.0395	0.72	Phenolic Acids
6	P-Coumaric Acid	$C_9H_{11}NO_3$	1.20	1.77	0.0213	0.81	Phenolic Acids
7	Glutathione	$C_{10}H_{17}N_3O_6S$	1.36	1.25	0.0289	1.62	Others
8	Citric Acid Monomethyl Ester	$C_7H_{10}O_7$	1.51	2.95	0.0068	1.95	Organic Acid
9	D-Pantothenic Acid	$C_9H_{17}NO_5$	1.62	1.81	0.0199	1.59	Organic Acid
10	Glucosyringic Acid	$C_{15}H_{20}O_{10}$	1.70	1.99	0.0371	0.62	Phenolic Acids

NO.	Name	Formula	RT [min]	VIP	FDR	FC	
11	Parishin E	$C_{19}H_{24}O_{13}$	2.13	12.08	0.0472	0.76	Parishins
12	Xanthosine	$C_{10}H_{12}N_4O_6$	2.19	1.71	0.0190	0.24	Others
13	Parishin I	$C_{38}H_{50}O_{24}$	2.91	3.50	0.0002	3.12	Parishins
		JT vs BT					
1	L-Histidine	$C_6H_9N_3O_2$	1.14	1.68	0.0473	2.67	Amino Acid
2	L-Aspartic Acid	$C_4H_7NO_4$	1.15	1.87	0.0107	1.77	Amino Acid
3	Glutamic Acid	$C_5H_9NO_4$	1.15	3.27	0.0239	1.67	Amino Acid
4	L-Serine	$C_3H_7NO_3$	1.16	1.24	0.0705	1.75	Amino Acid
5	Benzyl Acetate	$C_9H_{10}O_2$	1.51	1.24	0.0265	0.54	Phenolic Acids
6	Citric Acid Monomethyl Ester	$C_7H_{10}O_7$	1.51	4.23	0.0082	0.47	Organic Acid
7	Glucosyringic Acid	$C_{15}H_{20}O_{10}$	1.70	1.21	0.0521	1.60	Phenolic Acids
8	Phenylalanine	$C_9H_{11}NO_2$	1.75	2.99	0.0481	1.55	Amino Acid
9	Tryptophan	$C_{11}H_{12}N_2O_2$	2.66	2.27	0.0429	0.51	Amino Acid
10	Parishin I	$C_{38}H_{50}O_{24}$	2.91	4.17	0.0002	0.48	Phenolic Acids
11	1-O-Trans-Cinnamoyl-Beta-D-Glucopyranose	$C_{15}H_{18}O_7$	5.15	1.49	0.0050	0.31	Phenolic Acids
